# Oncology patients overwhelmingly support tissue banking

**DOI:** 10.1186/s12885-015-1416-5

**Published:** 2015-05-17

**Authors:** Jamie Bryant, Rob Sanson-Fisher, Elizabeth Fradgley, Timothy Regan, Breanne Hobden, Stephen P. Ackland

**Affiliations:** 1 Health Behaviour Research Group, Priority Research Centre for Health Behaviour, University of Newcastle & Hunter Medical Research Institute, HMRI Building, University of Newcastle, Callaghan, New South Wales Australia; 2 Hunter Cancer Research Alliance, Hunter Cancer Biobank, Hunter Medical Research Institute, University of Newcastle and Calvary Mater Newcastle Hospital, Waratah, NSW 2298 Australia

**Keywords:** Neoplasm, Living donors, Tissue banks, Informed consent

## Abstract

**Background:**

Translational biomedical research relies on the availability of human tissue to explore disease aetiology and prognostic factors, with the objective of developing better targeted treatments. The establishment of biobanks poses ongoing ethical considerations in relation to donors. This is a quantitative study exploring medical oncology patients’ preferences for contributing to tissue biobanks.

**Methods:**

The objectives of this study were to explore oncology patients’ preferences about tissue banking, including: 1) willingness to donate; 2) factors influencing donation decisions; 3) preferences about the use of donated tissue including permission systems, data linkage, and communication about research findings to donors. A cross-sectional survey was conducted in two tertiary oncology outpatient clinics. Eligible patients were approached by volunteers to complete a touchscreen survey in waiting rooms or while receiving intravenous therapy. Consenting participants completed demographic questions and received up to 12 previously validated items exploring preferences for donating tissue.

**Results:**

224 oncology outpatients participated over a ten month period (69.1 % consent rate; 64.4 % completion rate). Most participants were female (54 %), were a mean age of 62 years, and diagnosed with breast (26 %) and bowel (20 %) cancer. Most participants indicated willingness to donate tissue (84 %) and for their sample to be stored for future use (96 %). Participants preferred a blanket consent approach (71 %), samples to be linked to medical records (62 %) and for general results of the research (79 %) to be provided to them. Factors influencing willingness to donate tissue included personal (85 %) or familial health benefits (88 %) and a sense of duty to future patients (82 %).

**Conclusions:**

The overwhelming majority of oncology patients are willing to participate in a tissue bank, providing some support to explore ‘opt-out’ models of consent. To enhance patient acceptability, tissue banking programs should: (i) consider allowing blanket informed consent as well as opt-in models of consent; (ii) develop protocols allowing feedback of information about samples in line with patient preferences; (iii) provide clear information to potential donors about the benefits arising from donation.

## Background

Tissue banking involves the collection and storage of patient tissue samples for future biomedical research [1]. Banking of human tissue is an important tool for advancing translational research, allowing the study of genes, RNA and proteins to explore the biological mechanisms that underpin disease aetiology and biology, and the development of novel treatments [2]. In cancer, biobanking and the research that follows has capacity to facilitate more personalised therapies, with greater likelihood of benefit and fewer adverse effects. Given that patients who have been diagnosed with cancer may be more likely to experience some degree of benefit from biobanking, and can provide tissues of most interest to translational biomedical research, there is a critical need to understand potential donors’ willingness to participate in tissue banking, the factors influencing their decisions, and their preferences for how their samples are used.

Studies of the general public suggest a variable willingness to donate tissue for storage in a biobank, with rates ranging from 34 to 94 % [3–5]. In contrast, the willingness of patients with cancer to donate to biobanks is typically higher and less variable between studies, with rates ranging from 80 to 100 % [6, 7]. However, to date, research regarding the willingness of cancer patients to donate tissue is limited, largely qualitative, and confined to North America and Europe [4, 8, 9].

The factors affecting potential donors’ decision-making are unclear. Demographic factors including higher socio-economic status [3, 5], having any tertiary or college-level education [3, 10], being a patient [4], prior history of disease [10], higher levels of physical functioning [3], and previous experience providing samples [5, 10] have all been shown to be associated with greater willingness to donate. Few studies have explored more specific reasons that may influence patients’ decisions to donate tissue samples. For example, Lee and colleagues asked patients (a minority with a history of cancer) undergoing screening for breast cancer hypothetical questions regarding the donation of blood and saliva to a biobank [10]. Factors reported by respondents as influencing their decision to donate included feeling that donation would help patients with cancer (42.3 %) or advance science (34.6 %). Fewer considered benefit to themselves (15.3 %) or their family (13.7 %) as factors influencing their decision to donate. The majority of those that indicated they would decline the opportunity to donate cited privacy reasons (22.3 %) and no perceived personal benefit (19.6 %) as the main reasons. Patients with cancer have reported similar views, though this group has typically focused on altruistic motivations, as the most important factor when deciding to donate tissue [7].

Biobanks are intended to provide a resource to allow exploration of future as well as current research hypotheses. Research questions may require small or large sample sizes, and linkage to demographic and clinical data allows significant additional value, with an individual donation having the potential to be used for multiple projects over many years. This raises significant ethical considerations around informed consent for use of tissue samples [11–14], particularly as patients frequently provide enough tissue to cover the needs of several research projects [1, 12, 15]. The World Health Organisation [16] recommends that tissue banking studies request informed consent for all future research at the time of tissue donation, with the view that this blanket approval will reduce disruption to patients and increase the efficiency of translational research. However, a single overarching consent is not universally adopted by ethical governance boards or government policy [17]. Alternative consent processes that have been suggested include allowing patients to specify limits for sample use, and gaining informed consent for each project.

There is limited research about cancer patients’ preferences regarding what information is provided to them when requesting their consent for donation. A small study (n = 30) of patients with various cancer diagnoses found that 77 % would prefer to give blanket approval for use of donated samples, with only a minority of patients preferring to provide consent each time their sample was used [6]. A larger study (n = 100) of leukaemia patients reported that one-time blanket consent (59.6 %) was preferred compared to a tiered consent approach where patients could select the types of studies the sample could be used in (30.3 %), and re-consenting for each additional study (10.1 %) [18]. However, there is a need to explore the preferences of patients with a wider range of cancers.

The linking of stored samples to medical records raises the prospect of delivering relevant individualised information back to biobank contributors, which is appealing to most biobanking participants [5], and is associated with increased willingness to participate in biobanking among the general public [19, 20]. However, the specific views of cancer patients about linking to medical records remain unclear. There is also limited knowledge regarding patients’ preferences for receiving information about research studies that have utilised their tissue samples. Master and colleagues [18] reported that most patients with cancer preferred that the information be passed on to themselves and their doctor. However, they did not report on patients’ preferences for the level of detail provided. For example, individuals may wish to receive only detailed information resulting from their specific tissue sample donation; only grouped and non-specific results generated by all samples within the research project; or both sets of information.

A greater understanding of the factors that influence patient decisions to donate tissue to abiobank and preferences for the use of their donated samples is critical to the development of appropriate and acceptable biobanking protocols in Australia.

### Aims

This study aimed to examine medical oncology patient preferences about tissue banking, including:

- Willingness to donate tissue to be used in research;
- Factors influencing decisions to donate tissue; and
- Preferences about the use of donated tissue, including (i) the type of patient permission required for use; (ii) linking of tissue samples to identifiable medical records; and (iii) communication of findings from research using tissue samples back to donors.

## Methods

### Design

A cross-sectional survey was conducted with patients attending two tertiary medical oncology outpatient clinics in New South Wales, Australia.

### Participant eligibility

Eligible patients were: (i) able to read English; (ii) aged 18 years of age or older; (iii) attending a participating oncology outpatient centre for a consultation with a healthcare provider or to receive intravenous chemotherapy; and (iv) able to provide informed consent.

### Recruitment

Participants were approached by a trained research volunteer in the waiting room or treatment area of the medical oncology outpatient clinics. Volunteers provided a scripted overview of the study. Only general information on survey content was provided to reduce the potential to bias individuals’ decision to participate. Eligible patients were given a study information sheet and invited to complete the survey while waiting for their appointment or receiving treatment. Participants could pause or end the survey if they were called into their appointment before completion with the option of approaching the volunteer after their appointment to continue. Research volunteers kept a log of the number of patients who agreed and declined participation to allow determination of consent rate. Reason for non-participation was not recorded. Ethics approval was provided by the Hunter New England Human Research Ethics Committee (HNEHREC 12/08/15/4.04) and the Newcastle Private Medical Advisory Committee.

### Data collection

Surveys were administered via a tablet computer. Questions about tissue banking were part of a larger multi-site study which included three survey modules (in order of presentation): (i) the Consumer Preferences Survey where respondents identified possible changes that could improve their personal experience of care in the outpatient clinic {Fradgley EA, 2014 #25}; (ii) willingness to participate in future research, where respondents indicated their willingness to be contacted about future research opportunities; and, iii) willingness and preferences for tissue banking. Only items related to tissue banking will be reported here. In the first 14 months of data collection, medical oncology participants received all three survey modules. After this time, the Consumer Preferences Survey module was removed and participants received the remaining two modules. The biobanking questions were always presented last.

#### Demographic details

Participants self-reported gender; date of birth; marital status; highest level of education obtained; Aboriginal and/or Torres Strait Islander status; private health insurance coverage; and possession of a healthcare or concession card (which entitles the holder to government subsided medical care).

#### Disease and treatment characteristics

Participants were asked to self-report: the site of their primary cancer; time since receiving their diagnosis; the reason for their attendance at the outpatient clinic at the time of survey completion; and their frequency of clinic attendance over the previous 3 months.

#### Willingness and preferences for tissue banking

Twelve items exploring patient preferences for participating in tissue banking were selected from measures administered in previous studies [21]. These items were developed by Cousins and colleagues [22] and Kettis-Lindblad and colleagues [23] and have been tested in general populations and donor samples to ensure items are valid, relevant, and clearly worded.

All participants were asked whether they were willing for tissue left over from surgery to be used in research approved by an ethics committee (response options: yes; depends on the type of tissue; no; unsure). Those who agreed to donate samples, indicated it would depend on type of tissue, or were unsure, were considered as potentially willing to donate. Only those participants who were potentially willing to donate were asked whether they would allow remaining left-over tissue to be stored for future research (response options: yes; no; unsure). These participants also received the following questions: (1) *Factors influencing decisions to donate tissue.* Participants answered five items assessing the factors that would influence their decision for tissue to be used for research (response options: yes; no; unsure). (2) *Preferred permission:* Participants were asked to indicate their opinion about which system of consent should be used for stored tissue from three options: i) a system where the person must provide consent for each separate study their sample will be used in (i.e., repeat consent); ii) a system where the person gives permission once for all current and future studies (i.e., blanket consent); iii) a system where the person can choose either repeat or blanket consent. Participants were also asked how they would personally prefer to be asked for permission (response options: prefer to be asked each time; prefer to give permission once; unsure). (3) *Linking of tissue samples to identifiable medical records.* Participants were asked whether they would prefer a sample they had given to be linked to their medical record; unlinked; or no preference. Participants were told that “linked” meant their sample is given a code number that is confidential and only available to research staff, which can be linked to their name or medical records. Participants were told that ‘unlinked’ meant their sample is not given a number, therefore it is anonymous and cannot be traced back to their name or medical records. Participants were told that linked samples are of much greater value. (4) *Communication of findings from research using tissue samples back to donors.* Participants answered one item about whether they would prefer general information regarding the results of studies their tissue sample was used in to be reported back to them (response options: yes; no; unsure). All participants were asked if they had ever been asked for permission to use a tissue sample for research following a routine or planned medical procedure (response options: yes; no; unsure). For those that had been approached, participants were asked to indicate if they have given permission (response options: yes; no; unsure).

### Data analysis

Categorical measures were summarised using frequencies and percentages. Continuous measures were summarised using means and standard deviations.

## Results

### Consent rate

Of 504 medical oncology outpatients approached to participate over a 10 month period from March to December 2013, 348 agreed to complete the survey (69.1 % consent rate). A total of 224 patients (64.4 % of the total sample recruited) completed demographic items, disease and treatment items, and indicated preferences for tissue banking participation, and were included in the analysis.

### Demographic, disease and treatment characteristics

Participants were an average of 62 years old (SD = 14); females were slightly over-represented (56 %) (Table 1). The majority of participants were married or living with a partner (69 %), did not have private health insurance (56 %), and possessed a health care or concession card (64 %). A small proportion of the sample indicated they were of Aboriginal and/or Torres Strait Islander origin (2.2 %). The most common primary cancer types were breast (26 %) and bowel (20 %) (Table 2). The most common reported reasons for attending the clinic were related to a diagnosed cancer, with 29 % of participants attending for a routine exam and 54 % attending to receive tests or treatments. The majority of participants (51.6 %) indicated they had attended the clinic on a monthly or bimonthly basis in the preceding three months.

**Table 1:** Sample demographic characteristics (n = 224^*^)

	Mean	SD
Age	62	14
	n	(%)
Gender (n = 223)		
Male	98	44.0
Female	125	56.0
Marital Status (n = 224)		
Single or never married	24	11.0
Marred or living with partner	155	69.0
Separated or divorced	28	13.0-
Widowed	17	7.6
Highest level of education (n = 222)		
High school (year 10/school certificate or lower)	101	45.0
Higher school certificate	24	11.0
Diploma or trade certification	52	23.0
Bachelor degree	30	14.0
Post graduate degree	15	6.8
Aboriginal and/or Torres Strait Islander origin (n = 223)		
Yes	5	2.2
No	218	98.0
Private health insurance coverage (n = 224)		
Yes	99	44.0
No	125	56.0
Health care card (n = 224)		
Yes	144	64.0
No	80	36.0

^*^All columns do not sum to 224 due to missing data

**Table 2:** Sample disease and treatment characteristics (n = 224^*^)

	Number	(%)
Site of primary cancer (n = 210)		
Breast	54	26.0
Bowel	43	20.0
Blood (haematological)	23	11.0
Prostate	13	6.2
Gynaecological	14	6.7
Head and neck	5	2.4
Lung	19	9.0
Melanoma	2	1.0
Other	32	15.0
Don’t know	5	2.4
Time since diagnosis (n = 210)		
Three months or less	37	18.0
Four to six months	28	13.0
Six to twelve months	40	19.0
More than 12 months	49	23.0
More than three years	56	27.0
Reason for attendance at clinic (n = 222)		
For a regular check up	64	29.0
To discuss symptoms for an undiagnosed cancer	13	5.9
To discuss symptoms for an diagnosed cancer	25	11.0
To receive tests or treatments for a diagnosed cancer	120	54.0
Frequency of attendance at clinic in last 3 months (n = 224)		
First time at clinic	20	8.9
First visit in last 3 months, but attended within last 12 months	27	12.0
One previous visit	29	13.0
Two-three previous visits	52	23.0
Four to five previous visits	48	21.0
Six previous visits	17	7.6
Seven or more previous visits	31	14.0

^*^All columns do not sum to 224 due to missing data

### Willingness for tissue to be used in research

The majority of participants (84 %) indicated they would allow their left-over tissue to be used in research approved by an ethics committee, while 2.7 % reported their decision would depend on the type of tissue and 5.8 % were unsure (Table 3). Moreover, 96 % of participants who indicated that they would allow left-over tissue to be used also indicated they would allow remaining tissue used in the initial study to which they provided consent to be stored for future use. Thirty-five participants (17 % of the total sample) reported having been asked for permission to use their tissue sample for research following a medical procedure in the past, and all reported that they provided permission for their sample to be used.

**Table 3:** Participant willingness for use of tissue samples

	Number	(%)
Use of left-over tissue (n = 224)		
Yes	188	84.0
No	17	7.6
Unsure	13	5.8
Depends on tissue type	6	2.7
Storage of left-over tissue (n = 192*)		
Yes	185	96.0
No	2	1.0
Unsure	5	3.0

*Branching for responses of ‘No’ to first question “If asked, would you allow your left-over tissue to be used in a research study approved by an ethics committee?” means that 17 participants skipped the following question regarding storage of left-over tissue. An additional 15 participants did not provide a response for the question regarding storage of left-over tissue

### Factors influencing decisions for tissue to be used

Possible benefits to the health of family in the future (88 %), to the individual’s own health (85 %), and benefit to future patients (82 %) were factors the majority of participants indicated would influence their decision to participate in tissue banking (Table 4). Eighteen percent of participants were concerned that refusing to donate tissue might negatively affect the care they received and/or their relationships with healthcare staff.

**Table 4:** Factors influencing participant decisions for use of tissue samples (n = 182^*^)

	Yes n (%)	No n (%)	Unsure n (%)
Sense of duty to take part for the benefit of future patients (n = 182)	149 (82.0)	18 (9.9)	15 (8.2)
Possible benefits to own health (n = 176)	149 (85.0)	14 (8.0)	13 (7.4)
Possible benefits to health of family in the future (n = 181)	160 (88.0)	12 (6.6)	9 (5.0)
Concern refusal would negatively affect relationship with doctors or nurses (n = 172)	14 (8.2)	140 (82.0)	18 (11.0)
Concern refusal would negatively affect health care provided (n = 170)	12 (7.1)	140 (82.0)	18 (11.0)

^*^All rows do not sum to 182 due to missing data

### Preferences for use of tissue

#### Permission

Of the 194 participants who provided data regarding permission, 71 % preferred a system where individuals only need to be asked once for permission for use of their stored samples. Twenty-one percent of participants preferred a system where consent was sought each time the sample was used, and 8.2 % believed that the individual should decide what type of permission they would like to provide.

#### Linking with medical records

Of the 195 participants that provided data regarding linkage, approximately two-thirds (62 %) indicated a preference for their sample to be linked with their medical records, 29 % had no preference and 9.2 % preferred their sample to be unlinked.

#### Feedback of research results

Of the 165 participants that provided data regarding feedback of results, 79 % wanted to receive general information about the results of research in which their tissue sample was used. Fourteen percent of participants did not want to receive information and 7 % were unsure.

## Discussion

Despite the critical role tissue banking plays in advancing biomedical science and translational research, little research has specifically explored cancer patient perspectives on contributing to tissue banks. To our knowledge, this study is one of only a few which have quantitatively examined the views of patients with cancer, and the first to explore the views of Australian patients. This study explored patients’ willingness to donate tissue, the factors that influence decisions to donate, and patient preferences for providing consent. Understanding patient preferences about these issues has the potential to facilitate consent processes and the development of acceptable protocols for communication from tissue banking programs to donors.

Consistent with oncology patient preferences from qualitative work [6], the majority of patients in this study (84 %) indicated they were willing for their tissue to be used in an approved research project, of which 96 % indicated they would allow any leftover tissue to be used in future research projects. Based on our study, the willingness of Australian patients with cancer to donate to biobanks appears slightly higher than that of the Australian general public (75 %), although clear comparisons are difficult due to sample and questionnaire differences [19]. This might reflect a greater level of altruism in cancer patients: 88 % reported a desire to contribute to their family’s future health and 82 % a sense of duty to the wider community. It is important to note however that stated willingness to donate to biobanks is not necessarily reflected by actual donation. Pairwise comparisons between attitude surveys and actual participation rates from biobanking organisations are often discordant, with some biobanks reporting greater donation than willingness, and others vice versa. For example, among participants at a breast cancer screening clinic, Lee and colleagues [10] reported that 66 % indicated they would be willing make a donation, but only 56 % actually donated material. In the present study, all participants who indicated they had previously been approached to donate tissue had consented suggesting intention to donate and actual donation may be equivalent in this patient group. The high willingness to donate provides some support to explore the potential of ‘opt-out’ models of consent, which should provide a more cost-effective and time efficient method of tissue banking. The small proportion of patients (5.8 %) who remain unsure about donating tissue suggests a need for future research to explore the reasons for this.

Almost 20 % of participants reported feeling that non-participation might compromise their relationships with their doctor. This suggests that some patients will likely need reassurance from their physician that both their options for treatment and the quality of care provided will not be influence or compromised by their decision to participate or not participate in tissue banking. Patients and their healthcare providers therefore must have appropriate time and resources to clearly discuss the requirements and processes involved in tissue banking [1, 24]. There have been concerns expressed that clinical and hospital staff may be too busy to provide appropriate information regarding tissue donation, leaving patients unsure as to what they are consenting to, and how their sample might be used [1]. Future research might consider the provision of appropriate resources to assist in the discussion of biobanking and to ensure patients make informed decisions about donation.

Consistent with others studies [8, 18], the majority of participants (71 %) reported a preference for universal consent for all future research using their donated tissue sample [8, 18]. However, a greater proportion (21 %) of participants in the current study preferred to re-consent each time, only 10 % of participants preferred to re-consent that reported by Master and colleagues [8]. This is likely due to the questions used. Master and colleagues included an option for ‘tiered consent’. That is, patients were given a list of the types of studies in which donated samples could be used, as well as the type of health information that could be used. Tiered consent might provide greater certainty for patients that their samples will only be used in a way that they are comfortable with. Moreover, 58 % of participants in the Master and colleagues study felt that re-consenting each time was a waste of time and resources, although participants also reported that re-consenting would increase feelings of control, trust, and respect with the research team.

The majority (62 %) of participants indicated a preference for their donated sample to be linked to their medical records. That only 62 % were happy to have their sample linked to their medical records suggests that the additional benefits of medical record linkage have not been communicated clearly to potential donors. In addition, most participants (79 %) indicated an interest in receiving general information regarding the results of any study their sample was used in. Medical record linkage and patient preferences for the provision of specific information about their donated sample to be reported back to health care providers should be explored more closely. Both issues have ethical implications with respect to maximising the number of samples that are donated rom patients with cancer. The use of additional educational and decision support resources might be of benefit in this context.

Overall, the majority of patients with cancer appear willing to donate left over tissue to biobanks. These findings suggest that to enhance patient acceptability, tissue banking programs should:

1. Consider allowing patients to provide one-time, blanket informed consent, which was preferred by participants. Additional opt-in consent processes (e.g., a tiered consent approach) should be explored more closely in future research;
2. Develop protocols that allow for feedback to donors that is in line with patient preferences. Feedback about biobanking research projects and output might inspire donor confidence and stimulate further altruism;
3. Provide clear information to potential donors about who is likely to benefit from their decision to donate. This is includes highlighting that donors healthcare is unlikely to experience any direct benefit as a result of making a donation.

### Limitations

These findings must be considered in light of several limitations. First, participants were recruited from two tertiary medical oncology outpatient clinics in one regional area of Australia. Findings therefore may not represent the opinions and preferences of cancer patients across Australia. We recruited only a small sample of Aboriginal and Torres Strait Islander participants (2.2 % of the total sample), limiting the generalisability of our findings to this group. Second, the study was not adequately powered to explore variation by various disease and treatment factors. It is reasonable to assume there response might differ by factors including cancer type and stage, and treatment type and cycle. A larger sample is needed to explore these issues. Third, this study did not assess patients’ knowledge or understanding of the processes involved in donating to a biobank. Although the majority of participants were willing to donate left over tissue, differences in knowledge of and experience with biobanking might have biased participants responses to survey items.

## Conclusions

The vast majority of patients with cancer are willing to donate left over tissue to a biobanks, and are willing for that tissue to be used in future research projects. However, there appears to be a small proportion of patients who remain unsure. These findings suggest that to enhance patient acceptability, tissue banking programs should: (i) consider allowing blanket informed consent as well as opt-in models of consent; (ii) develop protocols that allow feedback of information about donated samples in line with patient preferences; (iii) provide clear information to potential donors about the benefits arising from donation.
